# Computed tomography guided needle biopsy: experience from 1,300 procedures

**DOI:** 10.1590/S1516-31802006000100003

**Published:** 2006-01-05

**Authors:** Rubens Chojniak, Rony Klaus Isberner, Luciana Marinho Viana, Liao Shin Yu, Alessandro Amorim Aita, Fernando Augusto Soares

**Keywords:** Needle biopsy, X-ray computed tomography, Neoplasms, Needles, Fine-needle biopsy, Biópsia por agulha, Tomografia computadorizada por raios X, Neoplasias, Agulhas, Biópsia por agulha fina

## Abstract

**CONTEXT AND OBJECTIVE:**

Computed tomography (CT) guided biopsy is widely accepted as effective and safe for diagnosis in many settings. Accuracy depends on target organ and needle type. Cutting needles present advantages over fine needles. This study presents experience from CT guided biopsies performed at an oncology center.

**DESIGN AND SETTING:**

Retrospective study at Hospital do Câncer A. C. Camargo, São Paulo.

**METHODS:**

1,300 consecutive CT guided biopsies performed between July 1994 and February 2000 were analyzed. Nodules or masses were suspected as primary malignancy in 845 cases (65%) or metastatic lesion in 455 (35%). 628 lesions were thoracic, 281 abdominal, 208 retroperitoneal, 134 musculoskeletal and 49 head/neck. All biopsies were performed by one radiologist or under his supervision: 765 (59%) with 22-gauge fine-needle/aspiration technique and 535 (41%) with automated 16 or 18-gauge cutting-needle biopsy.

**RESULTS:**

Adequate samples were obtained in 70-92% of fine-needle and 93-100% of cuttingneedle biopsies. The specific diagnosis rates were 54-67% for fine-needle and 82-100% for cutting-needle biopsies, according to biopsy site. For any site, sample adequacy and specific diagnosis rate were always better for cutting-needle biopsy. Among 530 lung biopsies, there were 84 pneumothorax (16%) and two hemothorax (0.3%) cases, with thoracic drainage in 24 (4.9%). Among abdominal and retroperitoneal biopsies, there were two cases of major bleeding and one of peritonitis.

**CONCLUSION:**

Both types of needle showed satisfactory results, but cutting-needle biopsy should be used when specific diagnosis is desired without greater incidence of complications.

## RESUMO

**CONTEXTO E OBJETIVO:**

Biópsia percutânea dirigida por tomografia computadorizada (TC) é amplamente aceita como um procedimento efetivo e seguro para diagnóstico em muitas situações clínicas. Sensibilidade, especificidade e acurácia dependem do sítio de biópsia e do tipo de agulha utilizada. Alguns estudos demonstraram vantagem no uso de agulhas cortantes em relação as agulhas finas tipo "Chiba". Este estudo apresenta a experiência de um centro de oncologia em biópsias guiada por TC.

**TIPO DE ESTUDO E LOCAL:**

Estudo retrospectivo realizado no Hospital do Câncer, A. C. Camargo, São Paulo, Brasil.

**MÉTODOS:**

1.300 biópsias consecutivas executadas de julho de 1994 a fevereiro de 2000 foram analisadas. A indicação foi presença de nódulo ou massa suspeita de neoplasia maligna primária em 845 (65%) casos e de metástase em 455 (35%). 628 biópsias foram realizadas em lesões torácicas, 281 abdominais, 208 retroperitoneais, 134 músculo-esqueléticas, e 49 na região de cabeça e pescoço. 765 (59%) biópsias foram executados com agulha fina tipo "Chiba" de 22 gauge com técnica de punção aspirativa e 535 (41%) com agulha cortante automática tipo "Tru-cut" de 16 gauge ou 18 gauge.

**RESULTADOS:**

Amostra apropriada foi obtida entre 70% e 92% das punções com agulha fina e de 93 a 100% das biópsias com agulha cortante. Diagnósticos específicos variaram entre 54 a 67% para agulha fina e de 82 a 100% para agulha cortante, dependendo do local da biópsia. Os índices de obtenção de amostra adequada e diagnóstico específico sempre foram melhores para a biópsia com agulha cortante. Em 530 biópsias pulmonares ocorreram 84 pneumotóraces (16%), e dois casos de hemotóraces (0,3%). Drenagem foi necessária em 24 casos (4,9%). Em biópsias abdominais ocorreram dois casos de hemorragia importante e um caso de peritonite; dois pacientes necessitaram cirurgia. Um implante tumoral no trajeto da biópsia foi observado em portador de carcinoma renal localmente avançado.

**CONCLUSÃO:**

Ambos os tipos de agulha mostraram resultados satisfatórios, mas, em nossa visão, agulha cortante deve ser utilizada quando um diagnóstico específico é desejado, sem levar a maior incidência de complicações.

## INTRODUCTION

Computed tomography guided biopsy has been widely accepted as an effective and safe procedure with high accuracy for diagnosis in many clinical settings.^1-6^ It is a simple way to obtain diagnoses in deep-seated lesions and guide therapeutic decisions, thus avoiding surgical biopsies in many instances. Fineneedle aspiration biopsy and, more recently, core biopsy have become the main choices for investigating many conditions.^1-6^ Computed tomography guidance is very versatile and can be used for various organs, from superficial soft-tissue lesions to deep-seated lesions in the thoracic or abdominal cavities. The sensitivity, specificity and accuracy also vary, depending on the target organ and the type of needle used. Some previous studies have demonstrated advantages in using cutting needles instead of fine needles, especially for diagnosing benign lesions.^5,7-11^ The choice of needle will depend on the patient's coagulation state, the characteristics of each lesion, the access route and the experience of both the radiologist and the pathologist.^1-12^

The complications depend on patient-related conditions, target organ, type of needle used and access route. Minor complications such as local discrete hemorrhage, pain and vasovagal reaction are not rare but are easy to deal with; mild complications such as pneumothorax are not rare in lung lesion biopsies but usually do not require drainage; major complications such as severe hemorrhage are rare and may even lead to death. Proper patient selection and technique can reduce the complication rates and, overall, percutaneous biopsy has a high benefit/risk ratio.^13^ The objective of this study is to present the results from a large series of computed tomography guided biopsies performed by the same radiologist, or by radiology residents under his supervision, at the Department of Radiology of Hospital do Câncer A.C. Camargo, an oncology center in São Paulo, Brazil. Our specific results for different sites and needles used are reported.

## METHODS

We retrospectively analyzed 1,300 consecutive computed tomography guided needle biopsies performed from July 1994 to February 2000 in 1,174 patients. All the biopsies accomplished during this period were evaluated. 728 (56%) of the 1,300 lesions were biopsied in male patients and 572 (47%) in female patients. The patients’ ages ranged from 0.3 to 98.0 years (mean: 59 years).

The biopsy sites were the following: 628 cases in the thoracic cavity (530 in the lung, 83 in the mediastinum and 15 in the pleura); 281 cases in the abdomen (195 in the liver, 54 in the pelvis and 32 in other abdominal sites); 208 cases in the retroperitoneum (66 aortocaval nodes, 54 in the kidney, 38 in the adrenals, 35 in the pancreas and 15 in the presacral space); 134 in the musculoskeletal system (45 in the thoracic wall, 36 in the paravertebral area, 27 in the limbs, 9 in the sacrum, 8 in the sternum and 9 in the superficial pelvic bones or muscles); 49 in the head and neck (27 in the orbit and 22 in the cervical area) (Table 1). The musculoskeletal lesions were all described together in one group because of their similar characteristics, such as being superficial and extracavitary. The clinical indication for the procedures was a focal lesion, nodule or mass that in all cases was suspected to be malignant (primary malignancy in 845 cases (65%) or metastatic lesion of a known cancer in 455 (35%).

**Table 1:** Biopsy site distribution among 1,174 patients studied

Lesion sites	Number of lesions	Subtotal
Thorax		628
Lung	530	
Mediastinum	83	
Pleura	15	
Abdomen		281
Liver	195	
Pelvic cavity	54	
Subphrenic space	30	
Gall bladder fossa	01	
Spleen	01	
Retroperitoneum		208
Aortocaval nodes	66	
Kidney	54	
Adrenal	38	
Pancreas	35	
Presacral	15	
Musculoskeletal		134
Thoracic wall	45	
Paravertebral	36	
Limbs	27	
Sacrum	09	
Pelvis	09	
Sternum	08	
Head and neck		49
Orbit	27	
Cervical	22	
**Total**	**1300**	**1300**

The patients’ preparation and lesion localization were the same for both types of needle used. The patients were admitted following six hours of fasting in preparation for the use of anesthetics and, if needed, intravenous contrast. Computed tomography scans were used to localize the lesion and to guide the insertion of the needle. Most procedures were performed under local anesthesia, but some younger and uncooperative patients required general anesthesia. The patients were positioned to allow the most direct access to the lesion: when the lesions were located in the thorax, passage through the aerated lung was minimized to reduce the risk of pneumothorax; when the lesions were mediastinal or abdominal, passage through large vessels and intestinal loops was avoided. Whenever necessary, intravenous contrast injection or changes in the patient's position were performed. Lesions were localized by means of contiguous scans of 5 mm in thickness. The best path was displayed on the monitor and electronic cursors were used to measure the distance from the skin and from a body marker. This information enabled calculation of the needle entry point, the degree of inclination and the distance from the entry point to the lesion. The entry point was localized on the patient's skin by correlation with the light beam from the computed tomography scanner. After positioning the needle, computed tomography scanning was done to confirm the position of the needle in relation to the lesion. The material was then collected and, after the procedure, all patients underwent computed tomography scans to evaluate the presence of possible complications (such as pneumothorax, hemorrhage and perforation of other structures).

In the cases of fine-needle biopsies, the 22-gauge "Chiba" needle was used with the aspiration technique, applying some firm back- and-forth movements and then withdrawing the needle under low negative pressure (to keep the collected material inside the needle and to avoid excessive hemorrhage). The slides obtained were then fixed in alcohol.

In the cases of cutting-needle biopsies, automated 16-gauge or 18-gauge side-cut needles were used and the automatic device was fired to advance the cutting edge. The fragments obtained were then preserved in 10% formalin solution.

In either case, the material obtained was examined by the pathology department staff at our institution, where they were routinely classified as adequate or inadequate for analysis and, when adequate, according to whether or not they provided a specific pathological diagnosis.

We reviewed the pathology reports and the patients’ records for all the procedures. All the reported information was collected, such as the indication for the biopsy, results and complications from the procedures. Finally, the success rate for diagnoses in different anatomical areas, in relation to the different types of needles and the biopsy complications detected, were described. The results from the different needles were compared by means of the Fisher and χ^2^ (chi-squared) frequency tests, and differences were considered significant when p < 0.05.

## RESULTS

The anatomical sites where the biopsies were performed are shown in Table 1. 765 procedures (59%) were performed using the 22-gauge fine-needle aspiration technique and 535 (41%) using automated 16-gauge or 18-gauge cutting-needle biopsy.

Out of the 765 fine-needle aspiration biopsies, 663 (87%) obtained material considered adequate for pathological analysis, while out of 535 cutting-needle biopsies, 503 (94%) obtained material considered adequate for analysis. Adequate samples were obtained from 70 to 92% of the fine-needle biopsies and from 93 to 100% of the cutting-needle biopsies. The specific diagnosis rate ranged from 54 to 67% for fine-needle and from 82 to 100% for cutting-needle biopsies, depending on the biopsy site.

The adequacy of the material and specific diagnosis rates obtained at different sites are shown in Tables 2, 3 and 4. Since the majority of the procedures were performed in the thoracic and abdominal regions, they have been presented separately, as have the lungs and liver in their respective categories. For any given site, the sample adequacy and the specific diagnosis rate were always better for the cutting-needle biopsy.

**Table 2:** Thoracic biopsies guided by computed tomography according to the needle type, sample adequacy rate and specific diagnosis rate

	Needle	n	Adequacy (%)	Specific diagnosis (%)
**Lung**	Fine	448 (85%)	392 (88%)	263 (67%)
	Cutting	82 (15%)	78 (95%)	64 (82%)
			
			p = 0.045	p = 0.001
	**Total**	**530**	**470 (89%)**	**327 (70%)**
**Thorax (except lung)**	Fine	38 (39%)	30 (79%)	14 (47%)
	Cutting	60 (61%)	56 (93%)	53 (93%)
			
			p = 0.055	p < 0.001
	**Total**	**98**	**86 (88%)**	**67 (68%)**

**Table 3:** Abdominal biopsies guided by computed tomography according to the needle type, sample adequacy rate and specific diagnosis rate

	Needle	n	Adequacy (%)	Specific diagnosis (%)
**Liver**	Fine	115 (59%)	106 (92%)	61 (58%)
	Cutting	80 (41%)	79 (99%)	69 (87%)
			
			p = 0.049	p < 0.001
	**Total**	**195**	**185 (95%)**	**130 (70%)**
**Abdominal (except liver)**	Fine	45 (52%)	40 (89%)	23 (58%)
	Cutting	41 (48%)	39 (95%)	37 (95%)
			
			p = 0.437	p < 0.001
	**Total**	**86**	**79 (92%)**	**60 (70%)**

**Table 4:** Biopsies guided by computed tomography according to needle type, sample adequacy rate and specific diagnosis rate

Site	Needle	n	Adequacy (%)	Specific diagnosis (%)
**Retroperitoneum**	Fine	62 (30%)	52 (84%)	28 (54%)
	Cutting	146 (70%)	136 (93%)	117 (86%)
			
			p = 0.038	p < 0.001
	**Total**	**208**	**188 (90%)**	**145 (70%)**
**Musculoskeletal**	Fine	20 (15%)	17 (85%)	9 (53%)
	Cutting	114 (85%)	103 (90%)	99 (97%)
			
			p = 0.439	p < 0.001
	**Total**	**134**	**120 (90%)**	**108 (81%)**
**Head and neck**	Fine	37 (76%)	26 (70%)	17 (65%)
	Cutting	12 (24%)	12 (100%)	12 (100%)
			
			p = 0.045	p = 0.001
	**Total**	**49**	**38 (78%)**	**29 (60%)**

Out of 530 lung lesions biopsies, 470 (89%) obtained a diagnostic sample. Fineneedle biopsy was used in 448 (85%) and cutting-needle in 82 (15%). The samples were considered adequate in 392 (88%) of 448 fine-needle biopsies and in 78 (95%) of the cutting-needle biopsies (p = 0.045). A specific diagnosis was obtained in 263 (67%) of the fine-needle samples and in 64 (82%) of the cutting-needle samples (p = 0.001).

In 195 hepatic lesion biopsies, 185 (95%) obtained diagnostic samples. Fine-needle biopsy was used in 115 (59%) and cuttingneedle in 80 (41%). The samples were considered adequate in 106 (92%) of the fine-needle biopsies and in 79 (99%) of the cutting-needle biopsies (p = 0.049). A specific diagnosis was obtained in 61 (58%) of the fine-needle samples and in 69 (87%) of the cutting-needle samples (p < 0.001).

The complications differed according to the location of the lesions and also with the type of needle used. Out of 628 thoracic procedures, pneumothorax was recorded in 76 (16%) of the 486 cases performed using fine needles and in eight (6%) of the 142 cases performed using cutting needles. Three cases of severe hemoptysis were reported, two of them with the use of fine needles. Two cases of hemothorax occurred, both with the use of fine needles. Thoracic drainage was necessary in 24 (4.9%) cases, all of them in procedures performed with fine needles.

There were four major complications (1.31%) reported among the 306 abdominal biopsies (203 intraperitoneal and 103 retroperitoneal). One case had a large splenic subcapsular hematoma in a pancreatic tail biopsy performed with a cutting needle. Another case consisted of loop perforation in a patient treated for a mixed ovarian cancer with surgery, chemotherapy and radiotherapy. This patient presented a persistently growing retroperitoneal tumor and the procedure was performed with a 22-gauge "Chiba" needle through an anterior approach. The third case had a large peripancreatic hematoma in a cutting-needle pancreatic biopsy, and the fourth case was a tumor implant in the abdominal wall after fine-needle aspiration biopsy for an advanced renal tumor.

## DISCUSSION

Needle biopsies can shorten the period of hospital stay and decrease the number of operations as well as the treatment costs.^1-6^

Computed tomography guided biopsy has been widely used as an effective and safe method for biopsy guidance, providing excellent visualization of the anatomy. It has great versatility and allows safe access to almost any deep-seated lesion in the human body. Through changing the patient's position and breathing techniques, the versatility and safety of the procedure can be further improved.^1,2,14,15^

Fine-needle biopsy has long been recognized as a useful technique for safely obtaining biopsies from deep-seated lesions. This technique has high sensitivity for diagnosing malignant disease.^1,3,5,9,11,12^ However, the quality of the tissue provided by cutting needles is superior, thereby allowing diagnoses to be made and further pathological investigations to be performed. Recent reports have described higher success rates from the use of cutting-needle biopsies, which have obtained adequate biopsy samples and have thus enabled accuracy in the diagnosis and specific histology results.^4,5,7-10^

The present study was conducted to evaluate the general results from CT guided biopsy in a large series, with evaluation of sample adequacy, diagnostic performance and major complications relating to CT guided biopsies routinely done in different body regions with the use of different types of needles in a oncology center in Brazil.

Our results confirm the advantages of cutting-needle biopsy in terms of adequacy of the sample and determination of specific diagnoses. Adequate samples were obtained using fine needles from 70 to 89% of the biopsies and from 93 to 100% for cutting needles, and the specific diagnosis rate ranged from 54 to 67% for fine-needle and from 82 to 100% for cutting-needle biopsies, depending on the biopsy site. These better performances for cutting-needle biopsy were statistically significant for most anatomical regions, except for the adequacy of the samples from non-pulmonary thoracic, musculoskeletal and non-hepatic abdominal lesions. In these situations, the numbers of cases and/or differences in the results were smaller. The reported success rate for obtaining adequate samples using needle biopsies ranges in the literature from 70 to 85% for lung lesions and from 80 to 95% for non-lung lesions.^1-12^ As expected, for any given site, the sample adequacy and the specific diagnosis rate were always better for cutting-needle biopsies. Fine-needle aspiration biopsy has long been recognized as useful because it is easily performed and provides high rates of diagnoses, especially for documentation of malignancy.^1,3,5,9,11,12^ More recently, studies have provided reassurances regarding the safety and better diagnostic performance of automated cutting needles, such that their use has increased, especially when a specific benign or malignant diagnosis is desired.^4,5,7-10^ In our results, the better performance of cutting-needle biopsy could be partially explained by the selection of different types of needle for different lesions based on our initial safety premises: there was a trend towards using fine needles for smaller, deeper and probably malignant lesions, especially in the lungs, and towards the use of cutting needles in larger, more outlying lesions. On the basis of our acquired experience and other reports, we now favor the use of cutting needles for most lesions, reserving fine needles mainly for patients with coagulation deficits.

Significant complications were reported descriptively due to the small number of cases.

The frequency of significant complications was in keeping with the literature. Most of them consisted of pneumothorax relating to lung lesions, with a low rate of tube place- ments.^1,9,13,15^ The reported incidence of pneumothorax in the literature ranges from 10 to 20%, with tube placement required for 5 to 10% of thesecases.^1,9,13,15^ All tube placements in our series were required after fine-needle aspiration biopsies but, again, there was a tendency to use fine needles for the difficult smaller and more central lung lesions, which could have increased the complication rate. The safety of the procedure in the abdomen and retroperitoneum was confirmed by the low incidence of major complications that occurred with either type of needle. The numbers of such complications were not enough to correlate with any specific needle type.^2,3,14,16-19^ A needle tract implant was observed in a patient with a locally advanced renal carcinoma. This type of complication is rarely reported in the literature and the risks from it are outweighed by the benefit of avoiding surgery in cases of advanced tumors and benign lesions.^20,21^ A greater concern is the potential seeding of initial, surgically curable malignancy: in such cases, despite the rarity of this complication, removal of the needle tract could be performed through surgery.^20,21^

Minor complications such as local mild pain or subcutaneous hemorrhagic collections may have been underestimated, since this was a retrospective study and these incidents might not have been recorded in post-biopsy subsequent evaluations.

## CONCLUSIONS

The present study has reported on the experiences of routine use of computed tomography guided biopsy in a Brazilian oncology center, with demonstration of the effectiveness and safety of the method. Overall, computed tomography guided biopsies have proven to be very useful, through obtaining diagnostic samples in most instances, with low incidence of complications, through providing guidance for therapeutic decisions and through avoiding surgery in many instances, especially in cases of diagnosing advanced malignant diseases. Both types of needles used showed satisfactory results but, in our view, cuttingneedle biopsy should be used when a specific diagnosis is desired without greater incidence of complications.
